# Prevalence of asthma and wheeze among preschool and school‐aged children in Africa: A meta‐analysis

**DOI:** 10.1002/puh2.199

**Published:** 2024-06-16

**Authors:** R. Mudau Rodney, K. Voyi Kuku, J. Shirinde Joyce

**Affiliations:** ^1^ Department of Human Nutrition Faculty of Health Sciences University of Pretoria Pretoria South Africa; ^2^ School of Health Systems and Public Health Faculty of Health Sciences University of Pretoria Pretoria South Africa

**Keywords:** asthma, asthmatic, children, prevalence, wheeze, wheezing

## Abstract

**Background:**

In Africa, asthma and wheezing are major health issues for children. There is a dearth of prior research examining the prevalence of asthma and wheezing in both preschool and school‐aged African children. Therefore, this meta‐analysis aimed to estimate the prevalence of asthma and wheezing in African infants and children aged 0 month to 8 years.

**Methods:**

We conducted a thorough electronic search of Academic Search Complete, MEDLINE, CINAHL, Scopus, Web of Science, PubMed, and Web of Science to find papers published between January 2012 and July 2023. We reviewed only research that was published in English. Independently, two review authors examined the studies, extracted the data, and evaluated the research studies. A fixed effects model and STATA 17 software were used. Using *I* ^2^, heterogeneity was assessed.

**Results:**

We considered 10 papers from Africa that examined the prevalence of asthma and/or wheezing in preschool and school‐aged children. Asthma prevalence ranged from 1.70% to 20.85% (*n* = 7 134 total participants), with a meta‐analysis showing an overall prevalence of 4.41% (95% CI: 3.95–4.87), with no heterogeneity (*I* ^2^ < 0). The historical prevalence rate of wheezing ranged from 4.71% to 67.72% (*n* = 8769 total participants), with a meta‐analysis revealing an overall prevalence of 22.91% (95% CI: 22.12–23.70), with no heterogeneity (*I* ^2^ < 0) and no significant differences observed between studies.

**Conclusions:**

Asthma and wheezing are prevalent among African preschool and school‐aged children, highlighting the need for comprehensive and localized research to address this public health issue.

## Graphical abstract

The burden of asthma and wheezing in this population group is substantial, emphasizing the importance of addressing this public health issue. Insufficient data on the prevalence of asthma and wheezing in specific African countries highlight the need for more comprehensive and localized research.

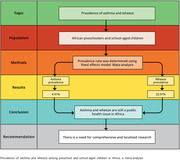

## INTRODUCTION

Asthma is a chronic inflammatory condition that impacts the respiratory airways, leading to variable symptoms including wheezing, shortness of breath, chest tightness, and/or coughing. Expiratory airflow limitation, which has the potential to resolve on its own or improve with pharmacological intervention, is what distinguishes the syndrome [1]. Several risk factors have been previously discovered in the literature, such as lower respiratory tract illnesses throughout early childhood, genetic predisposition, exposure to environmental allergens, as well as indoor and outdoor air pollution [2, 3].

Asthma can develop in both childhood and adulthood, although the development and severity of the condition vary between the two age groups. Asthma in children is more likely to start early, frequently before the age of five [4]. In addition to other characteristics including exposure to house dust mites, decreased lung function, genetic susceptibility, and bronchial hyperresponsiveness in children, early onset is linked to a higher chance of asthma persisting into adulthood [5]. Growth retardation is another possible side effect of asthma in children; however, it is transient and reversible [6].

Asthma may develop later in life in adults, usually between the ages of 20 and 40 [7]. The ultimate prognosis of asthma can be influenced by its intensity at onset; more severe individuals are less likely to experience total symptom management [7]. Exacerbations of asthma in adults can be brought on by stress, physical exercise, allergies, or air pollution exposure [8].

Prior studies have examined the prevalence of asthma and wheezing among children of school age and adolescents. The global prevalence of ever asthma and current wheeze in children aged 5–9 years was reported to be 17.79% and 11.77%, respectively [9]. In a study conducted by Song et al. [9] on the global, national, and regional prevalence of asthma in children of school age and adolescents, the study findings revealed that African regions exhibited the highest incidence of ever asthma (27.91%) and current wheeze (20.38%). Followed by the Western Pacific regions with a prevalence rate of ever asthma and current wheeze at 22.48% and 14.67%, respectively. Lastly, the regions of the Americas presented with a lower prevalence of both ever asthma (14.63%) and current wheeze (7.57%) as compared to the abovementioned regions.

The incidence of asthma in Africa has exhibited an upward trend over the last 20 years, with indications that its true prevalence may still be underestimated [10]. The long‐term impact of not diagnosing and adequately treating asthma early can have significant consequences on the quality of life of children and their caregivers (due to factors such as frequent waking up at night, wheezing, and hospital visits and admissions), as well as affecting personal relationships, emotions, and activities [11].

Asthma‐related fatalities, albeit few, warrant significant attention due to their avoidable nature [12, 13]. It is estimated that over 420,000 individuals worldwide succumbed to asthma, resulting in a daily average of over 1150 affected individuals [12, 14]. Over half of individuals reside in poor and middle‐income nations or marginalized communities [14].

However, there is a dearth of prior research examining the occurrence of asthma and wheezing in both preschool (from infancy) and school‐aged children within the African environment. Therefore, this meta‐analysis's aim and objective was to estimate the prevalence of asthma and wheezing in infants from 0 month to children 8 years of age in Africa.

## METHODS

The meta‐analysis was conducted in accordance with the guidelines outlined in the Preferred Reporting for Systematic Reviews and Meta‐Analyses statement [15]. Additionally, the study protocol has been registered in the International Prospective Register of Systematic Reviews under the number CRD42023447913.

### Eligibility criteria

This meta‐analysis focuses on the examination of observational and secondary studies conducted in Africa, specifically investigating the prevalence of asthma and wheezing in preschool and school‐aged children ranging from 0 month to 8 years of age. In children aged 0–8 years, early detection and treatment can help avert the asthmatic condition from becoming worse and enhance the child's quality of life. The study encompassed both community‐ and hospital‐based research. The researchers opted to omit studies of a qualitative nature, case studies, studies published in languages other than English, as well as studies published before the year 2012.

### Identification of studies for the review

An information specialist conducted an electronic search on the 14 July 2023 in Scopus, Web of Science, PubMed, MEDLINE, CINAHL, and Academic Search Complete for studies published from January 2012 to July 2023 using the following subject‐specific terms: (1) “Risk factors” OR “Contributing factors” OR “Predisposing factors” OR “Predictor” OR “Cause” OR “Prevalence” OR “Trends” (2) “Asthma” OR “Wheeze” OR “Wheezing” OR “Asthma symptoms,” (3) “Preschool” OR “early childhood” OR “kindergarten” OR “0–8 years” OR “School children,” (4) Africa/ or, (5) limit to (English language and year = “2012–Current” and children).

The principal investigator conducted a systematic search of the University of Pretoria Medical Library in South Africa, specifically targeting studies that were exclusively available in print format. The knowledge of an experienced information specialist facilitated this search. The reference lists of the articles were examined to identify any other research that met the eligibility criteria. The scope of the studies was restricted only to the English language.

### Adoption of the International Study of Asthma and Allergies in Childhood (ISAAC's) definition of asthma

International Study of Asthma and Allergies in Childhood (ISAAC) is a cooperative research network that seeks to provide a standardized methodology and promote worldwide cooperation to optimize the value of epidemiological research on asthma and allergy diseases. The ISAAC definition of asthma includes symptoms such as wheezing, coughing, and shortness of breath, as well as the usage of asthma medications [16].

Global Initiative for Asthma (GINA), on the other hand, is a global initiative that provides guidelines for the diagnosis, assessment, and management of asthma. GINA's definition of asthma focuses on the presence of symptoms, such as wheezing, cough, and shortness of breath, and the use of asthma medication, as well as the need for regular assessment and adjustment of treatment [17].

Although both ISAAC and GINA definitions share similarities, they differ in their emphasis and approach to asthma diagnosis and management. ISAAC's definition is more focused on the epidemiology of asthma and its prevalence in different populations, whereas GINA's definition is more focused on the clinical management of asthma and the need for regular assessment and adjustment of treatment [16, 17]. The current study aimed to determine the epidemiology of asthma and its prevalence in the African context.

### Study selection

Two reviewers, Rodney Mudau (RM) and Joyce Shirinde (JS), independently assessed the title and abstract results obtained from the search. This examination was conducted using Rayyan, an artificially intelligent program designed for systematic literature reviews. The researchers subsequently employed preestablished eligibility criteria to ascertain the suitability of publications for inclusion, ensuring that both reviewers were blinded.

The full texts of potential studies were acquired, in which at least one author of the review considered them appropriate for inclusion. The publications were subjected to a thorough evaluation process to determine their suitability for inclusion in the study. The researchers established contact with the authors of the study in instances where there was insufficient data or a requirement for additional elucidation. The disparities among the writers of the review were effectively addressed through joint deliberations.

### Data extraction and quality assessment

Two review authors, RM and JS, used a preestablished data extraction form to extract the data on asthma and wheezing in the past. The researchers collected data on the prevalence rates, as well as the overall number of individuals. The resolution of conflicts among the authors of the review was facilitated through the process of dialogue. The evaluation of study quality and risk of bias assessment was impeded as a result of the incorporation of various study designs, including secondary studies.

### Data synthesis and management

The quantitative data were aggregated in a statistical meta‐analysis utilizing the Statistics and Data (STATA) 17 software version. The researchers employed a fixed effects model to analyze cohort and case–control studies, and they evaluated heterogeneity by the application of the conventional chi‐square test. The fixed effects model was employed to counteract the significant heterogeneity demonstrated by the random effects model, which had an *I* ^2^ of 99.12%. A random effects model would have been advisable because the studies were small and very different. Thus, the effect sizes were calculated using fixed effects in each study, which solely accounts for within‐study heterogeneity. We sought to conclude only from the studies gathered for the synthesis. We assumed that these studies were not representative of a larger study population and were not drawn at random. A *p*‐value <0.05 was deemed to be statistically significant, indicating a strong level of confidence in the results. Similarly, a value of *I* ^2^ < 30% was indicative of homogeneity within the data [18].

### Ethical considerations (IRB statement)

The present study received approval from the University of Pretoria Research Ethics Committee (Ethics Number: 766/2019).

## RESULTS

### Search for studies

**FIGURE 1:**
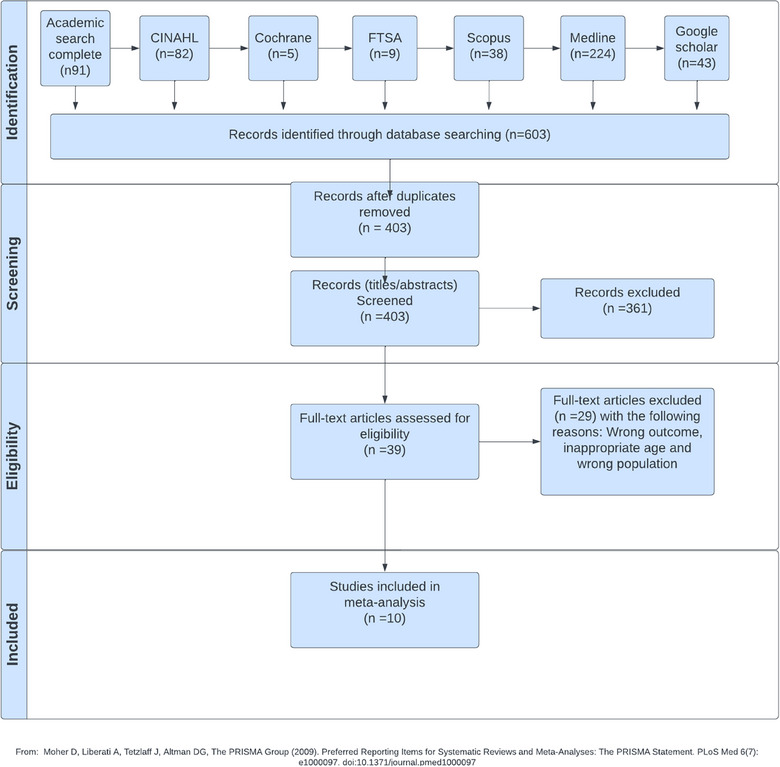
Flow diagram of the search, screening, and study selection for the systematic review (SR)/Meta‐analysis.

Figure 1 depicts the flow diagram that illustrates the sequential steps involved in the search results, screening, and selection of studies. A thorough examination of seven online databases yielded a total of 603 studies. Following the removal of duplicate entries, an aggregate of 403 studies was maintained. A total of 361 studies were excluded following the evaluation of titles and abstracts, resulting in a final selection of 39 studies for which full texts were obtained. A total of 29 studies were excluded from our analysis as a result of either not providing prevalence data or including an older age range. Consequently, our final dataset included 10 studies that specifically reported the prevalence of asthma and/or wheezing in previous periods. Therefore, two meta‐analyses pertaining to the prevalence of asthma and wheezing in the past incorporated studies that provided clear definitions for these specific conditions.

### Characteristics of included studies

Tables 1 and 2 present the respective characteristics of the studies that were used in the meta‐analysis on asthma and wheezing. We included five population‐based, three hospital‐based, one secondary analysis, and one direct method use studies from seven countries in Africa, across five geographical regions. Four studies were from the Southern Africa region: all four from South Africa [19, 20, 21, 22]. Three studies were from East Africa: one in Uganda [23], one in Tanzania [24], and one in Ethiopia [25]. One study was conducted in Northern Africa, specifically Morocco [26]. One study was conducted in West Africa, specifically Senegal [27] and lastly, one study was conducted in Central Africa, specifically Angola [28]. The population in the studies ranged from 280 to 3080 participants, and their ages ranged between 0 month and 8 years.

**TABLE 1:** Characteristics of studies included in the asthma meta‐analysis.

Reference	Country	Number of participants	Age in months	Asthma definition
[16]	Uganda	614	2–59	Asthma was defined according to GINA (Global Initiative for Asthma) guidelines which were modified by excluding the symptom of “chest tightness,” spirometry/peak expiratory flow measurements and by adding chest X‐ray findings to distinguish asthma from pneumonia
[19]	Morocco	1000	0–59	Asthma was defined as a physician diagnosis that usually consists of a careful history and physical examination. Moreover, they defined the number of all identified cases as the number of consultations for asthma in primary care
[17]	Tanzania	280	24–84	Caregivers were asked if the child had ever been diagnosed by a physician with asthma, and to report any of the following in the preceding year: wheezing, coughing without fever, shortness of breath, or chest tightness
[20]	Senegal	1513	60–96	Asthma was defined according to the International Study of Asthma and Allergies in Childhood (ISAAC) questionnaire: Has your child ever had asthma?
[21]	Angola	3080	72–84	Asthma was defined according to the International Study of Asthma and Allergies in Childhood (ISAAC) questionnaire: Has your child ever had asthma?
[14]	South Africa	647	0–41	Asthma was defined according to the International Study of Asthma and Allergies in Childhood (ISAAC) questionnaire: Has your child ever had asthma?

**TABLE 2:** Characteristics of studies included in the wheeze in the past meta‐analysis.

Reference	Country	Number of participants	Age in months	Wheeze in the past definition
[18]	Ethiopia	932	0–60	The following ISAAC questionnaire question was utilized in the study: “Has your child ever experienced wheezing or whistling in their chest?”
[20]	Senegal	1513	60–96	The following ISAAC questionnaire questions were utilized in the study: Has your child ever experienced chest wheeze or whistling before? Since the previous rainy season, has your youngster been wheezing or whistling in their chest?
[15]	South Africa	493	1–26	The following ISAAC questionnaire questions were utilized in the study: Mothers responded to the following questions with a yes or no answer: Has your child ever experienced chest wheeze or whistling? Have your kids ever experienced a dry cough in addition to coughs brought on by colds or chest infections?
[12]	South Africa	1143	0–24	Two binary outcome factors were taken into consideration: either the child had recurrent wheeze episodes (two or more wheeze episodes in a 12‐month period) or had at least one wheeze episode during the first 2 years of life
[21]	Angola	3080	72–84	The study employed questions from the ISAAC questionnaire: “Has your child wheezed or whistled in the chest in the last 12 months?” Parents were also asked about the number of wheezing episodes, impairment with sleep or speaking, relationship to physical activity, and bouts of nocturnal cough in the previous 12 months
[14]	South Africa	658	0–41	The following ISAAC questionnaire questions were utilized in the study: Has your child ever experienced wheezing or whistling in their chest? Wheezy chest sounds during or after physical activity
[13]	South Africa	950	0–60	We identified six wheeze indicators for each child: (1) Age at the first recorded episode; (2) age at the last recorded episode; (3) total number of distinct records across the observation period; (4) duration of the longest stretch based on the number of consecutive wheezing records; (5) total number of spells; (6) spell kind (0 indicates no wheeze, 1 indicates a single spell, and 2 indicates intermittent spells)

Abbreviation: ISAAC, International Study of Asthma and Allergies in Childhood.

### The risk of bias

Given that the number of studies is fewer than 30, it is evident that the statistical power of the funnel plot is significantly diminished, hence rendering it incapable of providing precise and reliable outcomes. Hence, the evaluation of publication bias and the methodological discrepancy was not possible. Moreover, it is crucial to acknowledge the potential presence of outcome bias, which refers to the selective reporting of certain outcomes by authors of a primary study, based on the direction and statistical significance of the data. The exclusion of studies conducted solely in English may result in the omission of pertinent and significant studies.

### The prevalence results

**FIGURE 2:**
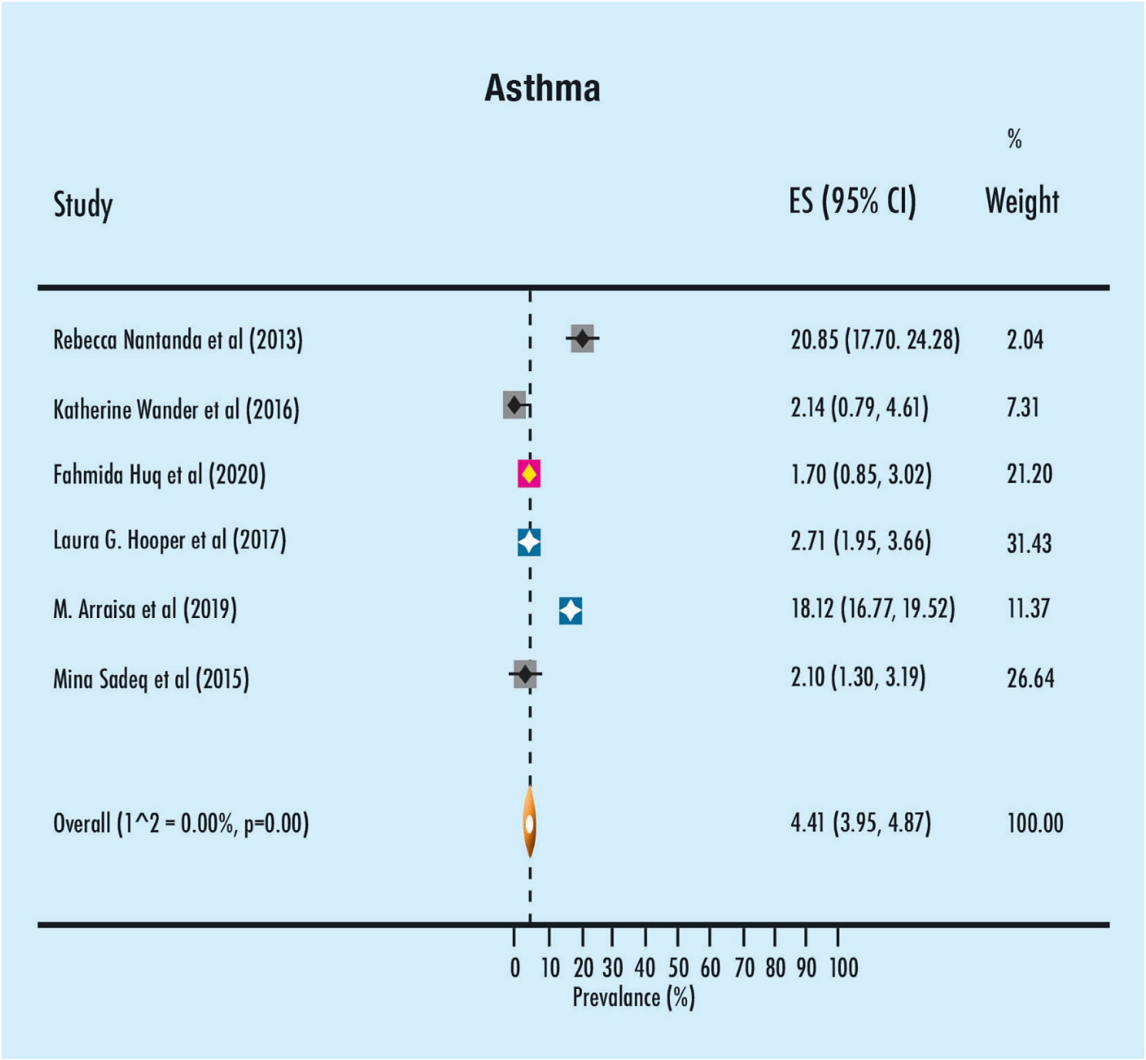
Meta‐analysis of the prevalence of asthma in the study population group.

**FIGURE 3:**
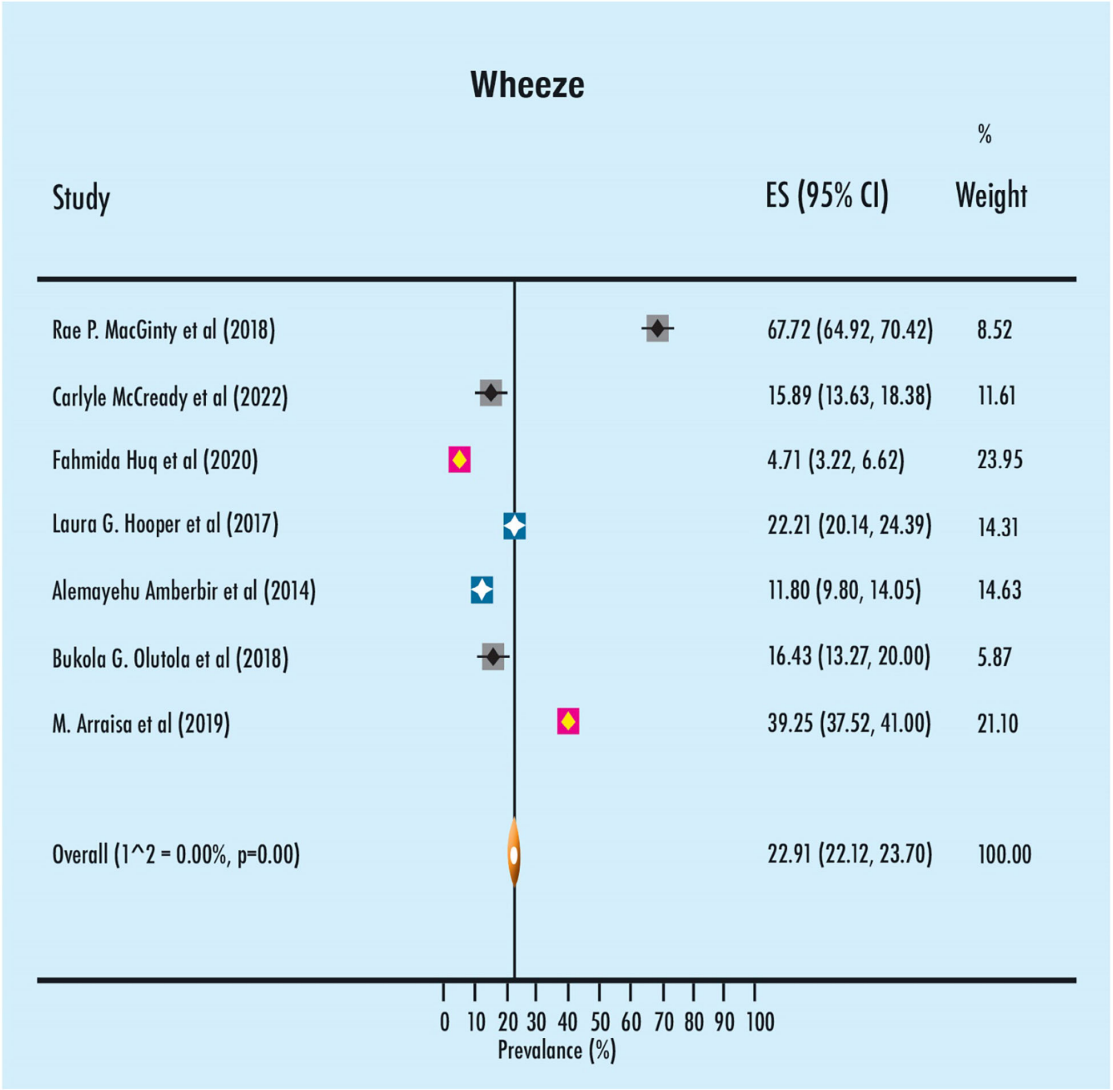
Meta‐analysis of the prevalence of wheeze in the past in the study population.

The findings from the meta‐analysis pertaining to the prevalence of asthma and wheeze in the past are displayed in Figures 2 and 3, respectively. The study found that the prevalence of asthma among children in the preschool and school‐aged groups was 4.41%, with a 95% confidence interval ranging from 3.95% to 4.87%. There was a lack of heterogeneity seen among the trials, as indicated by an *I* ^2^ value of less than zero. The occurrence rate of wheezing in the past among children in the preschool and school‐aged groups was found to be 22.91% (95% CI: 22.91–23.70). There was a lack of heterogeneity seen among the studies, as indicated by an *I* ^2^ value of less than zero.

## DISCUSSION

The current meta‐analysis has demonstrated that asthma and wheezing continue to pose a significant public health burden among preschool and school‐aged children in Africa. The prevalence of wheezing in the past had a higher incidence in comparison to the prevalence of asthma among this particular demographic.

The findings pertaining to the prevalence of asthma were slightly lower than anticipated, as it was lower than that of the African (27.91%) and global regions (17.79%) [9], but were within the anticipated range. Moreover, our findings were still lower than those reported by ISAAC phase 3 studies in children aged 6–7 in African and global regions [29, 30]. This observation aligns with the outcomes of a systematic review conducted by Adeloye et al., which examined the estimated prevalence of asthma in Africa [10]. The review indicated a notable upward trend in asthma prevalence across Africa in recent decades. Additionally, a comprehensive examination of existing scholarly works on the topic of childhood asthma in Southern Africa revealed that the occurrence of this condition exhibits variability but has shown an upward trend over the last 40 years. This trend is mostly pronounced in South Africa, where prevalence rates have been shown to range from 3.17% to 21.29% [31]. Furthermore, Kwizera et al. corroborated the aforementioned results by demonstrating that the occurrence of asthma in Sub‐Saharan Africa was estimated to be 4.6% among children under the age of five and 5.5% among children aged 5–14 [32].

Our study findings for the prevalence of wheeze in the past were high as compared to African and global prevalence studies conducted in this population group. Song et al. validated the abovementioned data, reporting a prevalence of 20.38% and 11.77% for both the African and global regions, respectively [9]. The above findings are further corroborated by the phase 3 ISAAC study, as they reported the prevalence of wheezing in African settings to be 10% and 14% in the 6–7 year and 13–14 year age groups, respectively [33]. Additionally, a research study conducted in Southern Africa revealed a 12‐month prevalence rate of wheezing and severe wheezing of 11.2% and 5.7%, respectively, among children aged 6–7 years. These rates were found to be lower in comparison to the findings of our study [31]. Furthermore, another study conducted in Gaborone, Botswana, showed a marginally elevated occurrence of wheezing within the past year, specifically among school‐aged children, with a prevalence rate of 16.5%. However, it is important to note that this prevalence remains lower than the prevalence observed in our study [34]. In addition, a meta‐analysis reported a prevalence of wheezing in infants in African countries at 15.97%, which was still below the prevalence rate in our study [35].

The African continent is facing an epidemiological transition characterized by a shift from communicable to noncommunicable diseases. The association between asthma and wheezing in African children has been linked to environmental exposures, including household air pollution and certain early‐life exposures that are prevalent in low‐ and middle‐income countries [33, 36].

Factors such as parental education, socioeconomic status, and urbanization have been linked to the prevalence of asthma and wheezing in African children. For example, children born or raised in urban areas and those with parents with higher education and socioeconomic status have been found to have a higher risk of asthma [10, 37]. Infections caused by human rhinoviruses (HRVs) have been identified as important triggers of wheezing in young African children. HRV‐induced wheezing during the first years of life has been associated with an increased risk of developing asthma in older children [20, 38].

The aforementioned findings underscore the intricate interaction among multiple risk factors that contribute to wheezing and asthma in African children. This underscores the necessity of implementing comprehensive approaches to tackle environmental, socioeconomic, and healthcare‐related factors to enhance respiratory health outcomes within this specific population. There are several challenges and disparities in the diagnosis and management of asthma in African children, including underrecognition and undertreatment [39, 40].

To the best of our knowledge, there is currently no existing meta‐analysis that provides an estimation of the prevalence of asthma and wheezing among infants and school‐aged children specifically within the African environment. Hence, this meta‐analysis exhibits a novel contribution. The absence of heterogeneity suggests that the meta‐analysis exhibits a high degree of robustness. Because there were so few studies in this meta‐analysis, it is possible that the true population was not adequately represented, which could lead to an under‐ or overestimation of the prevalence of wheezing and asthma. The lack of full uniformity of asthma and wheezing definitions among the included studies also contributes to the study's weaknesses.

## CONCLUSIONS

These findings indicate that asthma and wheezing are prevalent among preschool and school‐aged children in various African regions. The burden of asthma and wheezing in this population group is substantial, emphasizing the importance of addressing this public health issue. Insufficient data on the prevalence of asthma and wheezing in specific African countries highlights the need for more comprehensive and localized research.

## AUTHOR CONTRIBUTIONS

R. Mudau Rodney, J. Shirinde Joyce, and K. Voyi Kuku participated in the study's design, R. Mudau Rodney was involved in the data collection and statistical analysis, and R. Mudau Rodney, J. Shirinde Joyce, and K. Voyi Kuku were involved in interpreting the results and drafting and critically revising the manuscript. The published version of the work has been reviewed and approved by all authors.

## CONFLICT OF INTEREST STATEMENT

The authors declare no potential conflicts of interest concerning this article's research, authorship, and/or publication.

## ETHICS STATEMENT

The present study received ethical approval from the University of Pretoria Research Ethics Committee (Ethics Number: 766/2029).

## Data Availability

We did not receive ethics approval to share raw field data publicly. The data belong to the University of Pretoria (UP). The raw data analyzed in the current study are available from UP on reasonable request.
